# Supplementary material for: Modeling glioblastoma heterogeneity as a dynamic network of cell states
Source: Mol Syst Biol. 2021 Sep 16;17(9):e10105. doi: 10.15252/msb.202010105 (PMC8444284; doi:10.15252/msb.202010105)
Supplement: Supplementary file 6 — Source Data for Figure 5 [file MSB-17-e10105-s004.zip › Figure5A_sourcedata/GSEA_3071/hallmarks_stateI.GseaPreranked.1623742531165/HALLMARK_KRAS_SIGNALING_UP.html]

Details for gene set HALLMARK\_KRAS\_SIGNALING\_UP[GSEA]

|  || Dataset | state13071 |
| Phenotype | NoPhenotypeAvailable |
| Upregulated in class | na\_neg |
| GeneSet | HALLMARK\_KRAS\_SIGNALING\_UP |
| Enrichment Score (ES) | -0.23397228 |
| Normalized Enrichment Score (NES) | -1.1094093 |
| Nominal p-value | 0.2961832 |
| FDR q-value | 0.5148244 |
| FWER p-Value | 1.0 |
Table: GSEA Results Summary

  

Fig 1: Enrichment plot: HALLMARK\_KRAS\_SIGNALING\_UP      
 Profile of the Running ES Score & Positions of GeneSet Members on the Rank Ordered List

  

| PROBE | GENE SYMBOL | GENE\_TITLE | RANK IN GENE LIST | RANK METRIC SCORE | RUNNING ES | CORE ENRICHMENT || 1 | SPP1 |  |  | 0 | 2.808 | 0.0669 | Yes |
| 2 | CFH |  |  | 29 | 1.523 | 0.0951 | Yes |
| 3 | TFPI |  |  | 64 | 1.241 | 0.1148 | Yes |
| 4 | APOD |  |  | 76 | 1.195 | 0.1401 | Yes |
| 5 | NRP1 |  |  | 81 | 1.180 | 0.1671 | Yes |
| 6 | EMP1 |  |  | 122 | 1.012 | 0.1796 | Yes |
| 7 | INHBA |  |  | 140 | 0.941 | 0.1971 | Yes |
| 8 | TMEM100 |  |  | 170 | 0.872 | 0.2094 | Yes |
| 9 | ITGA2 |  |  | 194 | 0.839 | 0.2228 | Yes |
| 10 | KCNN4 |  |  | 327 | 0.648 | 0.2000 | No |
| 11 | LY96 |  |  | 451 | 0.547 | 0.1773 | No |
| 12 | EPHB2 |  |  | 468 | 0.535 | 0.1854 | No |
| 13 | GLRX |  |  | 485 | 0.524 | 0.1933 | No |
| 14 | CTSS |  |  | 509 | 0.502 | 0.1986 | No |
| 15 | BPGM |  |  | 546 | 0.481 | 0.1996 | No |
| 16 | ADGRA2 |  |  | 581 | 0.465 | 0.2008 | No |
| 17 | F13A1 |  |  | 607 | 0.448 | 0.2043 | No |
| 18 | PSMB8 |  |  | 669 | 0.423 | 0.1967 | No |
| 19 | CPE |  |  | 680 | 0.420 | 0.2038 | No |
| 20 | CROT |  |  | 739 | 0.400 | 0.1965 | No |
| 21 | KLF4 |  |  | 742 | 0.400 | 0.2055 | No |
| 22 | ITGBL1 |  |  | 784 | 0.389 | 0.2028 | No |
| 23 | BTBD3 |  |  | 838 | 0.369 | 0.1963 | No |
| 24 | ADAM17 |  |  | 890 | 0.353 | 0.1899 | No |
| 25 | EVI5 |  |  | 933 | 0.343 | 0.1859 | No |
| 26 | C3AR1 |  |  | 984 | 0.330 | 0.1793 | No |
| 27 | GPNMB |  |  | 1070 | 0.312 | 0.1621 | No |
| 28 | TRIB1 |  |  | 1134 | 0.301 | 0.1510 | No |
| 29 | GFPT2 |  |  | 1229 | 0.283 | 0.1305 | No |
| 30 | MMD |  |  | 1511 | -0.258 | 0.0551 | No |
| 31 | MYCN |  |  | 1556 | -0.262 | 0.0486 | No |
| 32 | HOXD11 |  |  | 1569 | -0.264 | 0.0514 | No |
| 33 | SPRY2 |  |  | 1612 | -0.269 | 0.0457 | No |
| 34 | GADD45G |  |  | 1657 | -0.275 | 0.0395 | No |
| 35 | PEG3 |  |  | 1784 | -0.292 | 0.0099 | No |
| 36 | SCG3 |  |  | 2025 | -0.322 | -0.0520 | No |
| 37 | ETS1 |  |  | 2086 | -0.332 | -0.0615 | No |
| 38 | ETV4 |  |  | 2325 | -0.378 | -0.1215 | No |
| 39 | ZNF639 |  |  | 2361 | -0.386 | -0.1225 | No |
| 40 | DCBLD2 |  |  | 2407 | -0.393 | -0.1262 | No |
| 41 | NIN |  |  | 2447 | -0.401 | -0.1279 | No |
| 42 | PLAT |  |  | 2501 | -0.415 | -0.1334 | No |
| 43 | GNG11 |  |  | 2554 | -0.427 | -0.1383 | No |
| 44 | HKDC1 |  |  | 2561 | -0.430 | -0.1298 | No |
| 45 | BMP2 |  |  | 2589 | -0.436 | -0.1272 | No |
| 46 | IL7R |  |  | 2665 | -0.460 | -0.1380 | No |
| 47 | SATB1 |  |  | 2870 | -0.527 | -0.1846 | No |
| 48 | TMEM158 |  |  | 2900 | -0.543 | -0.1800 | No |
| 49 | HDAC9 |  |  | 2927 | -0.560 | -0.1742 | No |
| 50 | RGS16 |  |  | 3134 | -0.708 | -0.2171 | No |
| 51 | G0S2 |  |  | 3184 | -0.762 | -0.2132 | No |
| 52 | CXCR4 |  |  | 3217 | -0.803 | -0.2033 | No |
| 53 | DUSP6 |  |  | 3227 | -0.816 | -0.1865 | No |
| 54 | KIF5C |  |  | 3232 | -0.823 | -0.1680 | No |
| 55 | GYPC |  |  | 3267 | -0.877 | -0.1569 | No |
| 56 | SOX9 |  |  | 3271 | -0.883 | -0.1368 | No |
| 57 | GPRC5B |  |  | 3326 | -0.974 | -0.1292 | No |
| 58 | PCSK1N |  |  | 3332 | -0.981 | -0.1073 | No |
| 59 | ETV5 |  |  | 3364 | -1.078 | -0.0906 | No |
| 60 | ID2 |  |  | 3374 | -1.110 | -0.0668 | No |
| 61 | IGFBP3 |  |  | 3433 | -1.384 | -0.0506 | No |
| 62 | AKAP12 |  |  | 3444 | -1.501 | -0.0177 | No |
| 63 | MAP3K1 |  |  | 3449 | -1.546 | 0.0180 | No |
Table: GSEA details [plain text format]

  

Fig 2: HALLMARK\_KRAS\_SIGNALING\_UP: Random ES distribution      
 Gene set null distribution of ES for **HALLMARK\_KRAS\_SIGNALING\_UP**
